# Assessment of Nutrients Intake in Pediatrics with Type 1 Diabetes and Dyslipidemia in Jordan

**DOI:** 10.2147/AHMT.S439046

**Published:** 2024-03-19

**Authors:** Reema Tayyem, Hala Nawaiseh, Sara Basem Zakarneh, Yasmen Khial, Sabika Allehdan

**Affiliations:** 1 Department of Human Nutrition, College of Health Science, Qatar University, Doha, Qatar; 2 Department of Nutrition & Food Technology, Faculty of Agriculture, The University of Jordan, Amman, 11942, Jordan; 3 Department of Biology, College of Science, University of Bahrain, Zallaq, Kingdom of Bahrain

**Keywords:** nutrients, type 1 diabetes, dyslipidemia, children, adolescents

## Abstract

**Background:**

Dyslipidemias are disorders of lipoprotein metabolism that occur during childhood and adolescence, often persist into adulthood, and increase the risk of developing atherosclerotic lesions. This study aimed to assess the potential association between nutrient intake and dyslipidemia in Jordanian pediatric patients diagnosed with type 1 diabetes mellitus.

**Methods:**

This cross-sectional study was conducted in Amman, Jordan, and involved 90 children and adolescents diagnosed with type 1 diabetes mellitus. Caregivers provided the following data: sex, age, type and dose of insulin, age at onset of type 1 diabetes, and level of physical activity. Anthropometric measurements were obtained using calibrated scales, and CDC growth charts were used to assess participants’ body weight status. Nutrient intake was estimated using a 120-item food frequency questionnaire (FFQ) previously validated in Jordanian children and adolescents. Serum lipid levels, including total cholesterol (TC), triglycerides (TG), high-density lipoprotein cholesterol (HDL-C), and low-density lipoprotein cholesterol (LDL-C), were measured. Binary logistic regression was used to assess the relationship between nutrient intake and dyslipidemia.

**Results:**

The results indicated that 36.7% of study participants had dyslipidemia. There were no significant differences in nutrient intake between dyslipidemic and normolipidemic individuals, except for a significantly higher median intake of vitamin B12 in the dyslipidemic group compared to the normolipidemic group (3.6 versus 2.7 µg, P-value = 0.046).

**Conclusion:**

This study found no significant association between the prevalence of dyslipidemia and nutrient intake in children and adolescents diagnosed with type 1 diabetes mellitus.

## Introduction

The prevalence of dyslipidemia has recently increased in the general population, including children. There is clear evidence that atherosclerotic plaque formation begins in the early years of life and in adolescence, leading to premature adverse cardiovascular problems. According to data from the National Health and Nutrition Examination Survey (NHANES), 20% of participants aged between 12 and 19 years were diagnosed with lipid disorders; however, the prevalence of dyslipidemia among children with obesity was 42% which was higher.^1^ Negative lifestyle habits, such as sedentism, low physical activity, and poor eating habits, play an important role in being overweight and obese, contributing to an increase in the prevalence of dyslipidemia.^2^ Dyslipidemia is the elevation of a group of blood lipids such as cholesterol, low-density lipoprotein cholesterol (LDL-C), triglycerides, and high-density lipoprotein cholesterol (HDL-C).^3^ Dyslipidemia is divided into two types: primary and secondary. Primary dyslipidemia is related to genetics, which means that it is inherited and is caused by a single mutation or multiple mutations that contribute to an elevation in triglyceride and cholesterol.^4^ Many factors are known to cause secondary dyslipidemia, including diabetes mellitus, cholestatic liver disease, nephrotic syndrome, chronic renal disease, hypothyroidism, obesity, smoking, excessive alcohol consumption, and some medications.^5^ Patients with type 1 diabetes mellitus (T1DM) are more 2–4 times likely to develop atherosclerosis than are those without diabetes. Moreover, the American Heart Association reported that children with type 1 diabetes have the highest risk of cardiovascular disease.^6^ In addition, both hyperglycemia and dyslipidemia are common in children and adolescents with T1DM, and most likely increase the risk of developing cardiovascular disease.^7^

Unhealthy eating habits are a significant risk factor for dyslipidemia.^8^,^9^ Diet has a similar effect on serum lipid levels in children and adults.^8^ Children’s blood lipid and lipoprotein concentrations were correlated with the type and amount of dietary fat consumed.^10^ It is important to note that no study has investigated the relationship between food intake and dyslipidemia in children in Jordan. Therefore, the purpose of this study was to evaluate nutrient intake and its association with dyslipidemia in pediatric patients diagnosed with T1DM in Jordan.

## Methodology

### Study Population

This cross-sectional study aimed to assess the nutrient intake of Jordanian children and adolescents with T1DM. Patients in this study were selected from the King Hussein Medical Center (Queen Rania Pediatric Hospital). A total of 90 Jordanian children and adolescents aged between 6 and 18 years who were diagnosed with T1DM from at least 1 to 10 years were selected. The inclusion criteria were an onset of disease of at least 1 year and less than 10 years, children and adolescents aged 6–18 years, insulin use, and Jordanian nationality. The exclusion criteria were patients who suffer from cancer, food allergies, autoimmune diseases, inability to communicate verbally, use of insulin pumps, and newly diagnosed T1DM. Ethical approval, obtained from the Institutional Review Board of the King Hussein Medical Center (Queen Rania Pediatric Hospital) under the number 1/2019/2863, adhered to the ethical standards outlined in the 1975 Declaration of Helsinki. Queen Rania Pediatric Hospital granted the researcher permission to utilize a private room with favorable physical conditions for conducting the interviews. The researcher explained the purpose of the study to all caregivers of the participants and required them to sign a consent form before data collection. All data for each participant in this study had an ID. All information was handled confidentially, and the researcher was the only one with access to the patients’ IDs.

### Data Collection

The data were gathered in a hospital setting. King Hussein Medical Center (Queen Rania Pediatric Hospital) provided care for children with T1DM. The outpatient department of the hospital served as the location for the data collection. Data such as sex, age, type, and doses of insulin were obtained from the caregivers of the participants using a personal information questionnaire. Face-to-face interviews with children’s caregivers were conducted for data collection between March 2020 and June 2022. Participants were divided into three groups according to their age into 3 groups, children (aged between 6–8 years), adolescents (aged–9-13), and pre-adolescents (aged–14-18). Additionally, they were divided according to their status as dyslipidemia (dyslipidemia group) or not having dyslipidemia (normolipidemic group).

#### Physical Activity Questionnaire

Physical activity levels were measured using a questionnaire. Children in grades 4 through 12 were given a general assessment of their level of physical activity using the Physical Activity Questionnaire for Older Children (PAQ-C) and Physical Activity Questionnaire for Adolescents (PAQ-A).^11^ The questionnaire evaluated the overall level of physical activity.^12^ These are self-administered, seven-day recall questionnaires that offer inexpensive, easy to administer, valid, and reliable assessment of physical activity for children and adolescents. The survey does not provide an estimate of calorie expenditure; detailed information on frequency, time, and intensity; or a distinction between different activity intensities, such as moderate and intense activities.^11^ Physical activity was recorded and presented as a score.

#### Dietary Assessment

The participants’ dietary information was gathered using a validated Arabic food frequency questionnaire (FFQ) with 120 items designed for children and adolescents.^13^ Participants were asked how frequently, on average, they had eaten each food item over the previous 12 months. Each FFQ question had two parts: a food list that was adjusted to be culturally appropriate for Jordanian children and teenagers’ food items, along with frequency responses for each item and portion size expressed in household measures (such as cups, spoons, and plates) and/or typical packing size. The food list was categorized into multiple groups based on food type. There were 10 options for frequency response: never, ≤ 12 years or less, 2–3 months, 1–2 weeks, 3–4 weeks, 5–6 weeks, 1 day, 2–3 days, 4–5 days, and ≤ 6 days. Foods typically consumed at specific times of the year were considered for seasonal variation. Food consumption frequency was designed to calculate intake throughout the year. Instead of using terms such as fruits, eggs, crackers, and pastries, common units or usual units were used for some foods such as one apple, one egg, and one piece of cracker or pastry. Food models were used to assist the participants in evaluating the consumed portion size of foods that could not be assessed using standard measuring units. Metric measurements in grams or milliliters were recorded for each food category. The FFQ also gathered data on the ways in which food was prepared and cooked, as well as on the use of particular oils, margarine, and butter. To estimate the daily intake of energy and nutrients, dietary data from the FFQ were evaluated using the dietary analysis software Food Processor program (ESHA Food Processor SQL version 10.9.0.0), with additional information on the types of foods consumed in Jordan.^14^

#### Anthropometric Measurements

Heights and weights of the children, pre-adolescents, and adolescents were measured. According to the body mass index (BMI)-for-age Centers for Disease Control and Prevention (CDC) growth charts, underweight, overweight, and obese values were classified as follows: underweight percentile < 5, healthy weight ≥5 and <85, overweight ≥85 and<95, and obesity ≥ 95.^15^ Body weight was measured while the participants wore only light clothing and no shoes. In addition, the subjects’ height was measured while standing without wearing shoes, their shoulders relaxed, and their arms hanging freely (to the nearest 0.5 cm).

#### Biochemical Tests

A trained lab worker collected blood samples from each participant, drawing 5 mL of blood with a 3-mL syringe and a simple tube filled with gel. Triglyceride (TG), total cholesterol, high-density lipoprotein cholesterol (HDL), and low-density lipoprotein cholesterol (LDL) levels were included in a group of blood tests called lipid profile parameters. Hemoglobin A1c (HbA1c) and fasting blood glucose (FBG) results were retrieved from the patients’ medical records.

### Statistical Analysis

Data were analyzed using SPSS (version 22.0; IBM SPSS Statistics for Windows, IBM Corporation). Descriptive analyses were performed to investigate the frequencies of various variables. The *Chi-square* test was used to determine the statistical differences between categorical variables, Chi-square was used. Data are displayed as the mean ±SD. The normality of the distributions of the dietary consumption variables was evaluated using the *Shapiro–Wilk* test. The differences in daily nutrient intake for both groups were reported as median (33.3th-66.7th) and determined using a non-parametric one-sample *T-test* (*Wilcoxon signed* ranked test) for variables that were not normally distributed. Age, sex, daily insulin dose (units/kg/day), type of insulin, BMI, and calorie intake were adjusted for in all multivariate analyses.^16^ To simplify the calculation of odds ratios and provide a better understanding of the clinical implications of our findings, all macronutrients and micronutrients were divided into tertiles. *P*-value < 0.05 was reflected as statistically significant.^16^ Multinomial logistic regression analysis was used to evaluate the associations between macronutrient intake, micronutrient intake, and dyslipidemia. The model was evaluated using three goodness-of-fit chi-square statistics performed with dyslipidemia as the dependent variable in the presence of one or more abnormal lipid and lipoprotein levels. The threshold values for total cholesterol were ≥ 200 for total cholesterol, ≥ 100 for TG in patients aged–6-9 years old and ≥130 for patients aged–9-19 years old, 130 for LDL cholesterol, and < 35 for HDL cholesterol.^17^ The National Cholesterol Education Program and Expert Panel on Cholesterol Levels in Children provided values for the plasma lipid and lipoprotein levels. Non-HDL-C levels in Bogalusa were equal to the LDL cutoffs for the National Cholesterol Education Program Pediatric Panel.^17^ Normolipidemic and dyslipidemia groups were formed in the study sample. P values for trends were assessed using a linear logistic regression test.

## Results

Table 1 presents descriptive, anthropometric, and biochemical information for the participants who were divided into two groups based on their lipid profiles. Participants (N= 90) were classified as dyslipidemic if there was at least one abnormal lipid profile variable (n= 33). Between the two dyslipidemic and normolipidemic groups, there was a significant difference in age groups (*P*=0.044). Additionally, statistically significant differences were observed in the incidence of disease (*P*=0.015), insulin type (*P*=0.041), morning insulin dose (*P*=0.007), and bedtime insulin dose (*P*=0.014). However, there were no statistically significant differences in weight (*P*=0.469), height (*P*= 0.326), and BMI (*P*=0.057). There were no significant differences in HbA1c and FBG levels between the dyslipidemia and normolipidemic groups (*P=*0.519 and *P=*0.474, respectively). The lipid profiles of diabetic children with dyslipidemia and those without dyslipidemia (mean SD and median) showed significantly higher levels of cholesterol (*P*=0.001), LDL (*P*<0.001), and TG (*P*<0.001). However, no statistical difference was detected in HDL levels (*P*=0.559).

**Table 1:** Descriptive, Clinical, Anthropometric and Biochemical for Participants with and without Dyslipidemia

Variable	Normolipidemic (n=57)	Dyslipidemic (n=33)	*P*-value
**Gender**	**n (%)**		
Male	21 (36.8)	19 (57.6)	0.056
Female	36 (63.2)	14 (42.4)	
**Age Group**			
Children	6 (10.5)	4 (12.1)	0.044
Pre-Adolescents	34 (59.6)	11 (33.3)	
Adolescents	17 (29.8)	18 (54.5)	
	**Mean ± SD**		
**Age (years)**	12.3±3.0	13.5±3.1	0.996
**Duration of diseases (years)**	3.9±3.2	4.0±2.5	0.015
**Physical activity level scoring**	2.0±0.51	1.96±0.53	0.795
**Anthropometric measurement**	**(Mean± SD)**		
Weight (kg)	46.3±17.6	47.9±17.5	0.469
Height (cm)	146.4±22.3	148.8±15.7	0.326
BMI category*	**n (%)**		
Underweight	1(1.8)	5(15.2)	0.012
Normal weight	40 (70.2)	13 (39.4)	
Overweight	11 (19.3)	11 (33.3)	
Obese	5(8.8)	4(12.1)	
**Types of insulin**	**n (%)**		
Rapid-acting	7(12.3)	0(0)	0.041
Pre-mixed	32(56.1)	17 (51.5)	
Long acting	0(0)	2(6.1)	
Rapid+ long	18 (31.6)	14 (42.4)	
**Doses of insulin**	**(Mean ±SD)**		
At morning	0.89±3.6	2.6 ±7.0	0.007
At breakfast	12.6±6.9	10.9 ±7.0	0.620
At lunch	14.1 ±7.2	12.6 ±7.9	0.626
At dinner	10.2 ±7.4	8.8 ±5.5	0.791
At bedtime	5.0 ±8.6	9.7 ±10.9	0.014
Total of insulin	42.7 ±19.9	44.5 ±19.9	0.450
**Biochemical tests**	**(Mean ± SD)**		
HbA1c (%)	10.0±2.3	9.8±2.8	0.519
FBG (mg/dL)	162.9±95.6	177.4±101.1	0.474
Cholesterol (mg/dL)	156.3±24.3	189.4±42.3	0.001
LDL (mg/dL)	85.4±18.9	109.2±41.3	<0.001
HDL (mg/dL)	60.1±13.0	48.2±16.4	0.559
TG (mg/dL)	74.3±20.2	207.1±122.9	<0.001

**Notes**: *BMI was classified based on BMI-for-age CDC growth charts: Underweight = BMI %< 5; healthy weight ≥5 and <85; overweight ≥85 and<95; obese ≥ 95.^15^ Data are presented as either percentages or means± standard deviation and a *P*-value<0.05 indicates statistical significance.

**Abbreviations**: BMI, Body mass index; HbA1c, Hemoglobin A1C; FBG, Fasting blood glucose; LDL, Low-density lipoprotein; HDL, High-density lipoprotein; TG, Triglycerides.

The macronutrient intakes of the normolipidemic and dyslipidemic groups are shown in Table 2. The median values for both the groups were similar. For the normolipidemic group, the median daily energy intake was 2451.7 (2157.2–2667.3) Kcals, while for the dyslipidemic group, it was 2428.9 (2032.0–2781.0) Kcals. There were no noticeable differences between any of the macronutrients (*P*=0.877). There was no statistically significant difference (*P*=0.461) between the dyslipidemia and normolipidic groups; however, the dyslipidemia group had a greater median energy from fat intake (kcal). In contrast, the normolipidemic group had a greater median carbohydrate (g) value, although the difference was not statistically significant (*P*=0.196). When compared to the normolipidemic group, The median daily consumption of cholesterol (mg) was not significantly higher in the dyslipidemic group as compared to normolipidemic group (253.2 (155.5–327.6) vs.196.0 (131.9–252.0), *P*=0.214).

**Table 2:** Daily Intake of Macronutrients of Normolipidemia and Dyslipidemia Participants with T1DM

Energy and Macronutrients	Normolipidemia (n= 57)	Dyslipidemia (n= 33)	
	Median (1st −3rd Tertiles)	Median (1st −3rd Tertiles)	*P*-value*
**Energy (kcal)**	2451.7(2157.2–2667.3)	2428.9(2032.0–2781.0)	0.877
**Fat (kcal/)**	976.4(777.4–1120.9)	1045.4(834.9–1234.0)	0.461
**Proteins (kcal)**	82.1 (69.3–95.2)	90.1(79.6–101.3)	0.236
**Carbohydrate (gm)**	294.8(239.1–322.8)	267.8 (238.1–294.7)	0.196
**Fibers (gm)**	29.8(26.3–34.5)	24.9(20.1–30.8)	0.082
**Soluble Fibers (gm)**	3.0(2.4–4.1)	2.5(2.2–3.2)	0.152
**Insoluble Fibers (gm)**	6.8(5.3–9.0)	6.1(4.8–6.9)	0.123
**Sugars (gm)**	68.5(53.0–96.0)	67.9 (49.8–77.1)	0.293
**Fat (gm)**	109.3 (86.6–124.9)	117.1(93.8–138.0)	0.464
**Saturated Fat (gm)**	24.0(18.1–27.4)	25.3(19.5–30.5)	0.549
**Monounsaturated Fat (gm)**	36.4 (26.9–46.8)	37.3(27.2–51.8)	0.660
**Polyunsaturated Fat (gm)**	20.8 (13.0–26.2)	25.1(15.3–33.0)	0.211
**Trans Fat (gm)**	0.26(0.23–0.34)	0.38(0.26–0.44)	0.130
**Cholesterol (mg)**	196.0(131.9–252.0)	253.2 (155.5–327.6)	0.214
**Omega-3 (gm)**	1.2 (0.96–1.4)	1.3 (0.99–1.4)	0.550
**Omega-6 (gm)**	18.7(11.7–23.9)	22.7(13.4–29.7)	0.214

Notes: *P-values were calculated as *Mann–Whitney U*-test. Data are presented as median (1st-3rd tertiles). A *P*-value<0.05 indicates statistical significance.

Regarding micronutrient intake of the normolipidemic and dyslipidemic groups, there were no significant differences between any of the medians in either group, except for vitamin B12 (Table 3). Table 3 shows that the dyslipidemic patients consumed significantly (*P=*0.046) more vitamin B12 than normolipidemic patients, with a median intake of 3.6(2.8–4.0) µg vs 2.7(2.1–3.5) µg, *P*= 0.046).

**Table 3:** Daily Intake of Micronutrients of Normolipidemia and Dyslipidemia Participants with T1DM

Micronutrients	Normolipidemia (n= 57)	Dyslipidemia (n= 33)	
	Median (1st −3rd Tertiles)	Median (1st −3rd Tertiles)	*P*-value*
**Vitamin A (µg)**	796.6(600.9–1017.3)	872.4 (618.8–1074.7)	0.624
**Vitamin D(µg)**	1.3 (0.79–2.4)	1.2 (0.97–2.3)	0.583
**Vitamin E (mg)**	12.8(8.6–15.6)	11.7(8.6–17.7)	0.867
**Vitamin K (µg)**	756.0(431.2–911.1)	808.0(249.8–877.1)	0.405
**Vitamin C (mg)**	153.8(115.6–194.3)	98.0 (83.1–150.0)	0.059
**Vitamin B1 (mg)**	1.6(1.4–1.8)	1.7(1.4–1.8)	0.950
**VitaminB2 (mg)**	2.0(1.5–2.3)	2.0(1.8–2.6)	0.379
**Vitamin B3 (mg)**	17.3 (15.5–20.5)	21.6(17.8–22.8)	0.168
**Vitamin B6 (mg)**	1.4 (1.2–1.7)	1.4(1.2–1.6)	0.675
**Vitamin B12 (µg)**	2.7(2.1–3.5)	3.6(2.8–4.0)	0.046
**Biotin (µg)**	26.9(19.2–32.9)	21.5(16.1–27.5)	0.152
**Folate (µg)**	434.5(380.2–520.2)	458.9(390.1–511.3)	0.851
**Folic Acid (µg)**	81.9 (64.3–107.6)	94.5(70.5–122.1)	0.264
**Pantothenic Acid (mg)**	5.1 (4.2–5.5)	5.3(4.3–5.8)	0.511
**Calcium (mg)**	1043.2(862.8–1285.3)	984.3(803.4–1233.5)	0.870
**Iodine (µg)**	86.7(68.1–112.2)	98.8(66.2–124.3)	0.741
**Iron (mg)**	33.9 (21.8–56.4)	25.9(19.3–52.4)	0.388
**Magnesium (mg)**	341.5(305.5–409.50	328.8(295.2–391.0)	0.522
**Phosphorus (mg)**	1088.6(882.1–1270.5)	1170.8(938.9–1336.3)	0.484
**Potassium (gm)**	3.3 (2.7–3.8)	3.1(2.5–3.5)	0.386
**Sodium (gm)**	2.5(2.1–3.0)	2.6(2.3–3.2)	0.511
**Selenium(µg)**	57.7(45.6–80.7)	72.3(53.0–83.0)	0.469
**Zinc (mg)**	8.9 (7.8–11.3)	10.5(8.3–12.3)	0.317
**Chromium (µg)**	2.9(2.5–3.7)	2.7(2.2–3.3)	0.309
**Copper (µg)**	1360.0(1190.0–1650.0)	1280.0(1163.2–1549.3)	0.541
**Manganese (mg)**	4.3(3.7–5.2)	3.6(3.2–5.5)	0.244
**Choline (mg)**	188.1(146.8–236.1)	188.6(161.1–255.3)	0.805
**Glycemic index**	54.0(51.2–55.2)	53.5(51.7–55.1)	0.933
**Glycemic load**	32.2(25.0–48.2)	38.0(27.0–47.1)	0.983

Notes: *P-values were calculated using *Mann–Whitney U*-test. Data are presented as median (1st-3rd tertiles). A *P*-value<0.05 indicates statistical significance.

Table 4 and Table 5 provide the odds ratios and corresponding 95% confidence intervals for each nutrient after being divided into tertiles depending on whether the study participants had dyslipidemia. Age, sex, daily insulin dose (units/kg/day), type of insulin, height, weight, BMI, presence of disease, and calorie consumption were adjusted using multivariate analysis. No significant association was found between dyslipidemia and nutrient intake.

**Table 4:** Association of Macronutrients Intake and Dyslipidemia Among Normolipidemia and Dyslipidemia Participants with T1DM

Energy and Macronutrients	OR* (95% CI) of Dyslipidemia			
	T1	T2	T3	*P*- trend**
**Energy**	1	0.88(0.25–3.08)	0.97(0.28–3.33)	0.980
**Fat**	1	0.87(0.19–3.94)	1.28(0.19–8.72)	0.856
**Proteins**	1	0.57(0.12–2.75)	0.61(0.8–4.95)	0.783
**Carbohydrate**	1	1.77(0.36–8.72)	1.77 (0.23–13.45)	0.779
**Fibers**	1	0.91 (0.26–3.15)	1.73 (0.40–7.53)	0.652
**Soluble Fibers**	1	1.80 (0.50–6.56)	1.12 (0.27–4.68)	0.598
**Insoluble Fibers**	1	2.53 (0.62–10.41)	1.69 (0.38–7.61)	0.422
**Sugars**	1	1.52 (0.39–5.98)	1.08(0.22–5.16)	0.783
**Fat**	1	0.87(0.19–3.94)	1.28(0.19–8.72)	0.856
**Saturated Fat**	1	1.02 (0.21–5.0)	1.51 (0.19–11.84)	0.859
**Monounsaturated Fat**	1	1.34 (0.35–5.16)	1.42 (0.30–6.69)	0.888
**Polyunsaturated Fat**	1	0.92 (0.23–3.72)	0.87(0.17–4.36)	0.986
**Trans Fat**	1	0.88 (0.25–3.17)	0.82 (0.21–3.17)	0.960
**Cholesterol**	1	1.05 (0.24–4.70)	0.99 (0.18–5.52)	0.995
**Omega-3**	1	0.92 (0.21–4.16)	0.72 (0.11–4.65)	0.926
**Omega-6**	1	0.88 (0.22–3.55)	0.94(0.18–4.86)	0.984

**Notes**: *OR and CI: odds ratio and confidence interval; OR was adjusted for age, sex, daily insulin dose (units/kg/day), type of insulin, height, weight, body mass index, occurrence of disease, and energy intake.^16^ **P- trend values were calculated using linear logistic regression test. A *P*-value<0.05 indicates statistical significance.

**Table 5:** Association of Micronutrients Intake and Dyslipidemia Among Normolipidemia and Dyslipidemia Participants with T1DM

Micronutrients	OR* (95% CI) of Dyslipidemia			
	T1	T2	T3	*P*- trend**
**Vitamin B1**	1	0.55 (0.13–2.39)	0.98(0.20–4.88)	0.631
**Vitamin B2**	1	0.76 (0.17–3.33)	0.56(0.11–2.95)	0.785
**Vitamin B3**	1	0.59 (0.10–3.50)	0.82 (0.09–7.04)	0.771
**Vitamin B6**	1	1.52 (0.36–6.48)	5.54 (0.93–33.01)	0.115
**Vitamin B12**	1	0.50 (0.11–2.20)	0.88 (0.21–3.65)	0.580
**Biotin**	1	1.03 (0.26–4.16)	1.20(0.25–5.82)	0.968
**Vitamin C**	1	0.81 (0.18–3.59)	2.18(0.45–10.52)	0.409
**Vitamin E**	1	0.72 (0.18–2.86)	0.68(0.15–2.99)	0.860
**Folate**	1	0.77 (0.18–3.28)	1.57(0.29–8.42)	0.601
**Folic Acid**	1	0.75 (0.20–2.74)	1.11(0.27–4.53)	0.809
**Vitamin K**	1	0.94(0.28–3.16)	1.12(0.30–4.28)	0.961
**Pantothenic acid**	1	1.06(0.22–5.16)	0.83 (0.14–4.85)	0.936
**Calcium**	1	1.04 (0.29–3.64)	1.53(0.37–6.32)	0.801
**Iodine**	1	0.72 (0.17–3.00)	1.05(0.25–4.46)	0.833
**Iron**	1	0.72 (0.20–2.64)	1.22 (0.34–4.42)	0.715
**Magnesium**	1	1.09(0.27–4.51)	2.17(0.33–14.04)	0.635
**Phosphorus**	1	1.43 (0.31–6.57)	2.58 (0.33–20.25)	0.644
**Potassium**	1	1.21 (0.29–4.98)	1.68(0.29–9.88)	0.839
**Selenium**	1	0.39 (0.08–1.95)	1.02 (0.23–4.52)	0.327
**Sodium**	1	1.29 (0.31–5.32)	0.82(0.14–5.02)	0.788
**Zinc**	1	0.94 (0.19–4.52)	1.25(0.20–7.89)	0.913
**Vitamin D**	1	1.31 (0.33–5.29)	1.82 (0.45–7.35)	0.689
**Vitamin A**	1	1.07 (0.31–3.61)	1.19(0.29–4.92)	0.971
**Chromium**	1	1.09 (0.24–4.89)	1.47(0.31–7.04)	0.854
**Copper**	1	1.02(0.28–3.74)	2.46 (0.42–14.34)	0.489
**Manganese**	1	0.81 (0.22–3.06)	1.46 (0.33–6.41)	0.664
**Choline**	1	0.84 (0.19–3.70)	0.84 (0.16–4.56)	0.972
**Glycemic Index**	1	0.77(0.22–2.70)	0.64(0.18–2.30)	0.785
**Glycemic Load**	1	0.62(0.17–2.32)	1.07(0.27–4.19)	0.678

**Notes**: *OR and CI: odds ratio and confidence interval; OR was adjusted for age, sex, daily insulin dose (units/kg/day), type of insulin, height, weight, body mass index, occurrence of disease, and energy intake.^16^ **P- trend values were calculated using linear logistic regression test. A *P*-value<0.05 indicates statistical significance.

## Discussion

Diabetes mellitus (DM) is an epidemic proportion.^7^ T1DM accounts for 5–10% of all DM cases of DM worldwide.^7^ The data indicate that T1DM has been augmented at an average rate of 3–4% per year in children and adolescents.^7^ Patients with T1DM have a 2–4 times greater risk of developing atherosclerosis earlier in life, leading to increased morbidity and mortality compared to people without DM.^18^ Moreover, cardiovascular events account for > 44% of the total mortality among these patients.^19^ Studies have reported serum lipid abnormalities in children with T1DM.^18^ An association between elevated HbA1c levels and serum lipid abnormalities.^18^,^20^

In this cross-sectional study, among Jordanian children and adolescents with T1DM, the prevalence of dyslipidemia was 37%. Several studies have indicated that the prevalence of dyslipidemia in children with T1DM varies between 29% and 66%.^2^,^21–23^ Our results are consistent with those of a study conducted among 202 children and adolescents with T1DM, in which 26.2% of the children had dyslipidemia.^7^ In addition, data from a study conducted in Turkey showed that the prevalence of dyslipidemia among adolescents with T1DM was 30.3%.^24^ The current data also agree with the findings of a case-control study that found that the prevalence of dyslipidemia was significantly higher among children and adolescents with T1DM (65%) than in the non-diabetic control group (28.2%).^25^

However, this is relatively lower than a retrospective study conducted among Egyptian children and adolescents with T1DM, which reported a prevalence of (70.47%).^7^ The wide range of dyslipidemia frequency among children with diabetes in various studies may be due to multiple genetic factors in different ethnic groups, local dietary habits, age ranges, and different reference ranges for dyslipidaemia.^2^,^23^

The current results showed that children with dyslipidemia had significantly higher levels of TGs, LDL-C, and total cholesterol (TC) than diabetic children with a normal lipid profile. This is in concordance with data from a previous study that found that among Egyptian diabetic children, hypertriglyceridemia was the predominant type of dyslipidemia and reported higher levels of HbA1c among untreated newly diagnosed children with T1DM than among diabetic children with good glycemic control.^26^,^27^ Also, have found that hypercholesterolemia was the most common type of dyslipidaemia and hypertriglyceridemia was the least common among diabetic patients.^28^ Data from a cross-sectional study conducted among patients with T1DM aged 2–18 years indicated that 67.3% had at least one abnormality in the serum lipid profile, and the predominant lipid profile abnormality was associated with elevated LDL levels (62% abnormality).^7^ These differences are usually related to different glycemic controls in various studies.^28^

According to data from the SEARCH for Diabetes in Youth (SEARCH) study, lipid abnormalities are more prevalent in children and adolescents with poor or suboptimal glycemic control.^29^ In addition, data from the SEARCH study reported that the total and LDL-C, TGs, non-HDL-C, and apolipoprotein B concentrations increased as HbA1c increased.^29^ Diabetic dyslipidemia is characterized by decreased HDL-C levels and increased LDL-C, and TGs.^24^ The American Heart Association has categorized children with TIDM as having the highest risk of cardiovascular disease and has recommended both lifestyle changes and pharmacological treatment for those with elevated LDL levels.^30^

The Global IDF/ISPAD Guideline for Diabetes in Childhood and Adolescence (2014) recommends screening for fasting blood lipids when DM is stabilized in children over 10 years of 10 years.^31^,^32^ Moreover, if there is a family history of hypercholesterolemia or early cardiovascular diseases (CVDs) or if the family history is unknown, screening should be started at the age of 2 years.^31^,^32^ However, if normal lipid profiles have been obtained, screening should be repeated every 5 years.^31^,^32^

The current findings indicate that there were no significant differences in HbA1c and FBG levels between diabetic children with dyslipidemia and those with normolipidaemia. Several studies have indicated that poor glycemic control is associated with elevated serum lipid levels.^22^,^33^ It has been suggested that glycemic control is an important modifiable risk factor that is associated with the development of dyslipidemia as well as in the development of hypertriglyceridemia.^33^ It has been found that long-term glycemic management predicts the presence of atherosclerotic plaques on ultrasonography even in the absence of symptoms of coronary vascular disease.^34^ Intensive insulin therapy has also been reported to improve cardiovascular events in patients with childhood-onset DM.^34^ A few mechanisms have been hypothesized to show a direct effect of hyperglycemia on gene transcription of coagulation factors.^35^

Data have indicated that Insulin is a natural antagonist of the platelet hyperactivity.^35^ It enhances endothelial generation of nitric oxide (NO) and prostacyclin (PGI2), and sensitizes platelets to PGI.^35^ Therefore, long-term insulin deficiency in patients with T1DM, in combination with poor glycemic control, dyslipidemia, and dysfunctional coagulation cascade, may provide a significant risk for developing dyslipidemia.^35^ Additionally, the current data indicated that there were statistical differences in the incidence of disease (*P-*value<0.05) between children with dyslipidemia and normolipidemic diabetes. The current data are inconsistent with several previous studies that have shown that there were no significant differences in the duration of DM between the dyslipidemic and normolipidemic groups.^22^,^36^ Dyslipidemia was observed among children and adolescents with T1DM regardless of the duration of diabetes.^22^,^36^

The current data agree with the results reported by Moayeri and Oloomi (2006), who found that lipid levels were positively correlated with DM duration of DM.^37^ Several studies have revealed that C-reactive protein is elevated during the first year of T1DM diagnosis, and both interleukin 6 and fibrinogen levels are elevated in individuals with an average disease duration of 2 years.^38–40^ The current results showed that there were no significant differences in macronutrient intake between diabetic children with dyslipidemia and those with normolipidemia. However, the dyslipidemia group had a greater median energy from fat intake. It has been demonstrated that the adherence to macronutrient recommendations was the most important aspect among youth with T1DM.^41^ Several studies have revealed that children and adolescents with T1DM consumed more fat and saturated fat than age-based recommendations, and more than healthy controls.^41–43^

Moreover, Patton (2011) found that the total percentage of energy from fat in children and adolescents ranged from 31% to 47%, which was higher than the Healthy People 2010 recommendation of less than 30%.^41^ In addition, the mean percentage of energy from saturated fat ranged from 11% to 15%, which was higher than the American Diabetes Association recommendation of less than 7%.^44^ Youth with T1DM consumed significantly less than the recommended number of servings of both fruits and vegetables per day.^45^ However, young children with T1DM reported a higher proportion of energy from vegetables (10% vs 6%) compared to controls and a similar proportion of daily energy from fruits (10% vs 12%) compared to controls.^42^

Low fiber consumption has been reported to significantly increase TC, TGs, and VLDL concentrations.^46^ This could be explained by decreased cholesterol absorption, decreased hepatic cholesterol synthesis, and decreased levels of circulating cholesterol due to increased cholesterol use, which is driven by intestinal bile acid sequestration by fiber.^46^ Moreover, it has been demonstrated that the Mediterranean-style diet has been improve dyslipidemia, in particular LDL-C, and reduce the risk of CVDs.^47^

The major strength of this study is the use of an ethnically validated FFQ. In addition, this study is one of the few Jordanian studies to assess dyslipidemia among children and adolescents with TIDM. Furthermore, the inclusion of data on physical activity, body weight, and dietary intake, all of which are known to influence lipid levels, is also a strength of this study. The current study had several limitations. First, it was limited by the lack of assessment of various apolipoprotein levels and carotid artery intima-media thickness, which are both significant predictors of CVD risk. Second, because of the cross-sectional nature of the study, repeated measurements of fasting lipid profiles were not performed over time among diabetic children and adolescents. Additionally, even though we have adjusted the OR for many confounding factors, a residual effect may still persist. Finally, the study sample size was small compared with other studies from countries with higher populations. Therefore, studies with larger sample sizes are required to shed more light on this topic.

In conclusion, this study demonstrated a relatively high prevalence of dyslipidemia in the study children and adolescents with T1DM. High TGs, LDL-C, and TC levels were the most frequent abnormalities observed in the dyslipidemic group. The study did not show any association between macro and micronutrients and dyslipidemia in the study participants. As dyslipidemia is a modifiable risk factor for CVD among children and adolescents with T1DM, longitudinal studies are needed to evaluate the effects of early diagnosis and treatment of dyslipidemia on the progression of complications. Moreover, annual screening for dyslipidemia in children and adolescents with TIDM is recommended. The current study supports the need for nutritional guidance for the treatment of dyslipidemia in this population.
